# DriVQA: A gaze-based dataset for visual question answering in driving scenarios

**DOI:** 10.1016/j.dib.2025.111367

**Published:** 2025-02-03

**Authors:** Kaavya Rekanar, John M. Joyce, Martin Hayes, Ciarán Eising

**Affiliations:** a Department of Electronic and Computer Engineering, University of Limerick, Limerick V94 T9PX, Ireland; b Esports Science Research Lab, Lero, The Science Foundation Ireland Centre for Software Research, University of Limerick, V94 T9PX, Ireland

**Keywords:** Eye-tracking, Human attention patterns, Attention mapping, Object tracking, Computer vision, Scene analysis, Autonomous driving

## Abstract

This paper presents DriVQA, a novel dataset that combines gaze plots and heatmaps with visual question-answering (VQA) data from participants who were presented with driving scenarios. Visual Questioning Answering (VQA) is proposed as a part of the vehicle autonomy trustworthiness and interpretability solution in decision-making by autonomous vehicles. Collected using the Tobii Pro X3-120 eye-tracking device, the DriVQA dataset provides a comprehensive mapping of where participants direct their gaze when presented with images of driving scenes, followed by related questions and answers from every participant. For each scenario, the dataset contains: images of driving situations, associated questions, participant answers, gaze plots, and heatmaps. It is being used to study the subjectivity inherent in VQA. Its detailed gaze-tracking data offers a unique perspective on how individuals perceive and interpret visual scenes, making it an essential resource for training VQA models that rely on human-like attention. The dataset is a valuable tool for investigating human cognition and behaviour in dynamic, real-world scenarios. DriVQA is highly relevant for VQA models, as it allows the systems to learn from human-like attention behaviour when making decisions based on visual input when trained. The dataset has the potential to drive advancements in VQA research and development by improving the safety and intelligence of driving systems through enhanced visual understanding and interaction. DriVQA has significant potential for reuse in various research areas, including the development of advanced VQA models, attention analysis, and human-computer interaction studies. Its comprehensive gaze plots and heatmaps can also be leveraged to improve applications in autonomous driving, driver assistance systems, and cognitive science research, making it a versatile resource for both academic and industrial purposes.

Specifications Table

**Table:** 

Subject	Computer Vision and Pattern Recognition
Specific subject area	Computer Vision and Pattern Recognition focuses on enabling machines to interpret, analyze, and understand visual data by identifying patterns and objects*.*
Type of data	Table, Image, Figure, Analyzed data
Data collection	The collection of eye gaze data in DriVQA was taken using an eye-tracker device (Tobii Pro X3–120). The images used in the experiment are all from the NuImages [2] dataset. There are two tests in the experiment designed. All 34 participants were asked the same questions prepared from the questionnaire template (questions and anonymised responses provided). The heatmaps and gaze plots were generated using the visualisations tool in Tobii Studio-3.4.5 software [10]. The 48 absolute heatmaps and gaze plots are provided, which show general attention patterns. All participants have at least 2 years of driving experience. Participants’ driving experience is provided in a separate sheet with their corresponding ID.
Data source location	Data collection Location: Esports Science Research Lab, Lero, Tierney Building, University of Limerick, Ireland. Data stored in Mendeley Data
Data accessibility	Repository name: DriVQA Data identification number: 10.17632/p25744hwrc.1 Direct URL to data: https://data.mendeley.com/datasets/p25744hwrc/1 Instructions for accessing these data: Click on the URL provided and the data is accessible for public in Mendeley Data.
Related research article	*None*

## 1 Value of the Data

• DriVQA is an open-source dataset available for VQA models with its focus completely on driving scenarios. Visual Questioning Answering (VQA) can improve vehicle autonomy trustworthiness and interpretability [[2], [3], [4]]. They are recently being explored in the context of autonomous driving to enhance the understanding of the environment through visual inputs and enable more intelligent decision-making by autonomous vehicles [[5], [6], [7], [8], [9]].
• This dataset is relevant for VQA models, as it allows the systems to learn from human-like attention behaviour when making decisions based on visual input in a driving scenario. The gaze plot and heatmaps data have the potential to guide VQA models in selecting the most relevant regions of an image for answering specific questions, much like a human would focus on key areas of a driving scene.
• DriVQA can be used to study the subjectivity inherent in visual question answering. The dataset's structure allows for the analysis of diverse human responses to the same driving scenario, highlighting how different individuals focus on various aspects of the image when answering questions.
• DriVQA offers potential for improving the accuracy and interpretability of VQA models, especially in the context of autonomous driving, driver assistance systems, and traffic safety research.
• DriVQA stands out from general eye-tracking datasets [[11], [12], [13], [14], [15], [16], [17]] by uniquely integrating detailed gaze data (plots and heatmaps) with a visual question-answering (VQA) framework specifically tailored for driving scenarios, enabling the study of human-like attention, subjectivity in perception, and decision-making in safety-critical contexts; its high-quality Tobii Pro X3-120 eye-tracking data, coupled with driving images and participant responses, provides a versatile resource for advancing autonomous systems, human-computer interaction, and cognitive science research, while enhancing the interpretability and trustworthiness of AI models in vehicle autonomy.

## 2 Background

In a previous study [1], the authors of this work demonstrated a filter that helps mimic human attention patterns significantly, laying the groundwork for improved VQA capabilities in autonomous driving. The limitation of this approach is that it was assumed that humans are accurately describing what they are observing, leaving scope for subjectivity in data. For this current work, to tackle the limitation of subjectivity, an eye-tracking device (the Tobii Pro X3-120 [10]), which delivers real-time data of where a person is looking on a screen, is used. By doing so, the human attention patterns in a driving scene is captured and can be used to refine the accuracy and objectivity of a VQA model.

## 3 Data Description

The dataset is divided into two folders: Test 1 and Test 2. Every participant has been given an ID (P01, P02, etc.). The corresponding gaze plots (folder named G) and heatmaps (folder named H) can be found in the respective participants’ folders. The absolute heatmaps and gaze plots are found in separate folders for both tests (Absolute Heatmaps and All_Gaze Plots respectively). The responses recorded for every participant are available with the image and question asked in the .xlsx sheet (Responses) where every participant has a new sheet labelled with their respective IDs.

Here is a comprehensive list of everything available in the dataset:

i. Empty questionnaire (Questions.pdf): Contains the images and list of questions that the participants were asked
ii. Participants’ answers (Responses.xlsx): Contains the responses from every participant in a separate sheet labelled with their respective participant IDs.
iii. Gazeplots (Folder Gaze Plot): Each gaze plot represents the exact points of focus and their sequence on the driving images, with the size of these exact points illustrating the length of attention. These are available in the folder named ‘Gaze Plot’ for every participant.
iv. Heatmaps (Folders Heatmap): Available in the folders named ‘Heatmap’, heatmaps are used to illustrate the number of gaze points and their durations in various areas of the scene.
v. Coordinates data (Participant_id.xlsx): Available for every participant in their respective folders, this file provides comprehensive information like how long a participant focused on the object of interest, which eye was used, etc.
vi. Description of Variables used (Variable_Descriptions.pdf): Contains tables that detail the name of the variable, its format and a description of the variable's purpose categorically.
vii. Driving experience (Experience.pdf): Contains the years of driving experience for every participant in the experiment with their corresponding participant IDs.
viii. Images used with camera information (Folder Images): Contains all the images used for the experiment in their respective camera folders (e.g., the folder Back camera has 4 images, etc.).

## 4 Experimental Design, Materials and Methods

**Fig. 1:**
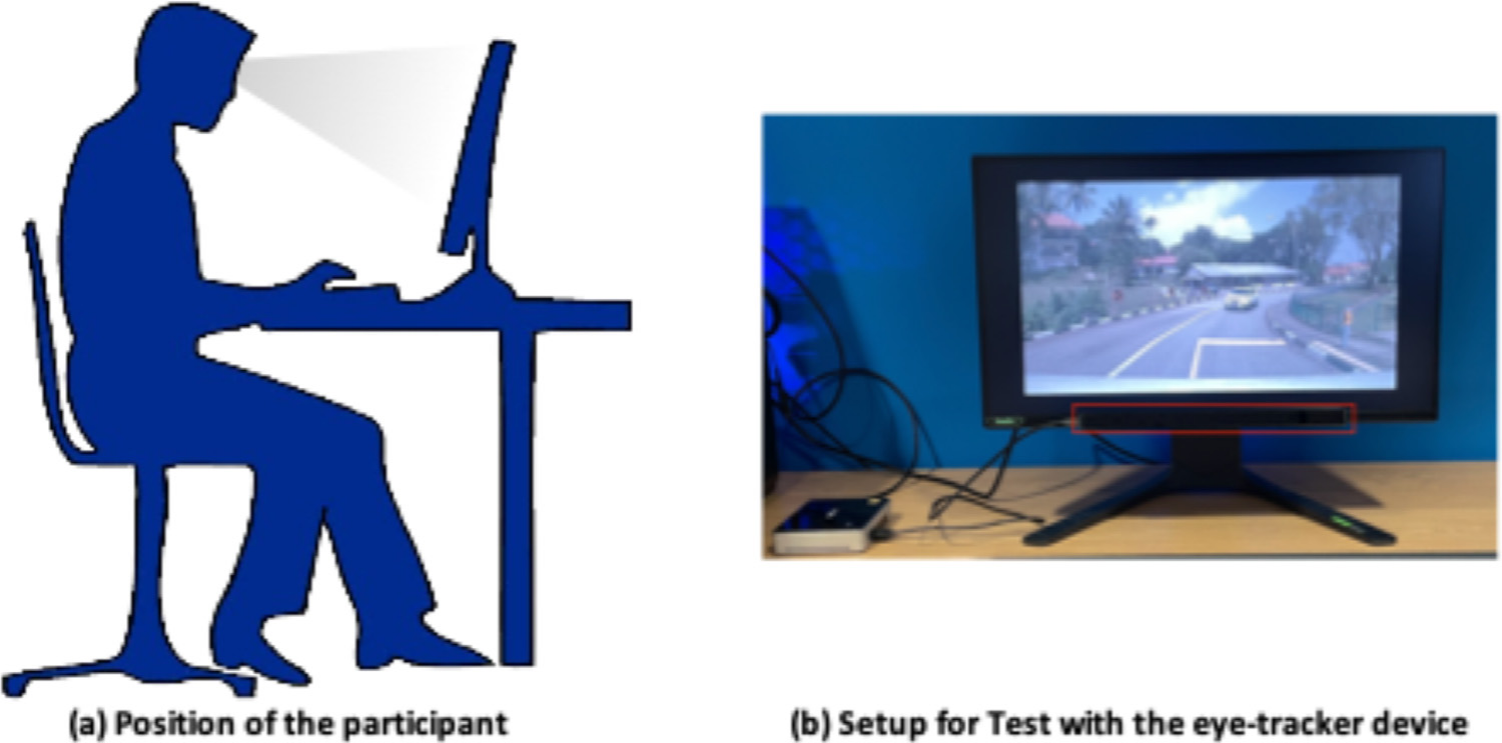
Experiment Setup. Fig. 1(a) shows the position of how the participant was seated during the test and Fig. 1(b) details on the setup used in terms of the equipment involved. The red rectangle box highlights the Eye-tracker device- Tobii Pro X3-120 and the screen shows the design of the experiment in a summarised view using TobiiProStudio-3.4.5 software.

The experiment conducted was designed to analyze the consistency between what participants report they looked at when answering a question in a driving scenario and what they focused on. The Tobii Pro X3-120 [10] was the eye-tracker device used. The software used to conduct the experiment was Tobii Pro Studio- 3.4.5 [10]. The setup can be seen in Fig. 1(a) and (b).

**Fig. 2:**
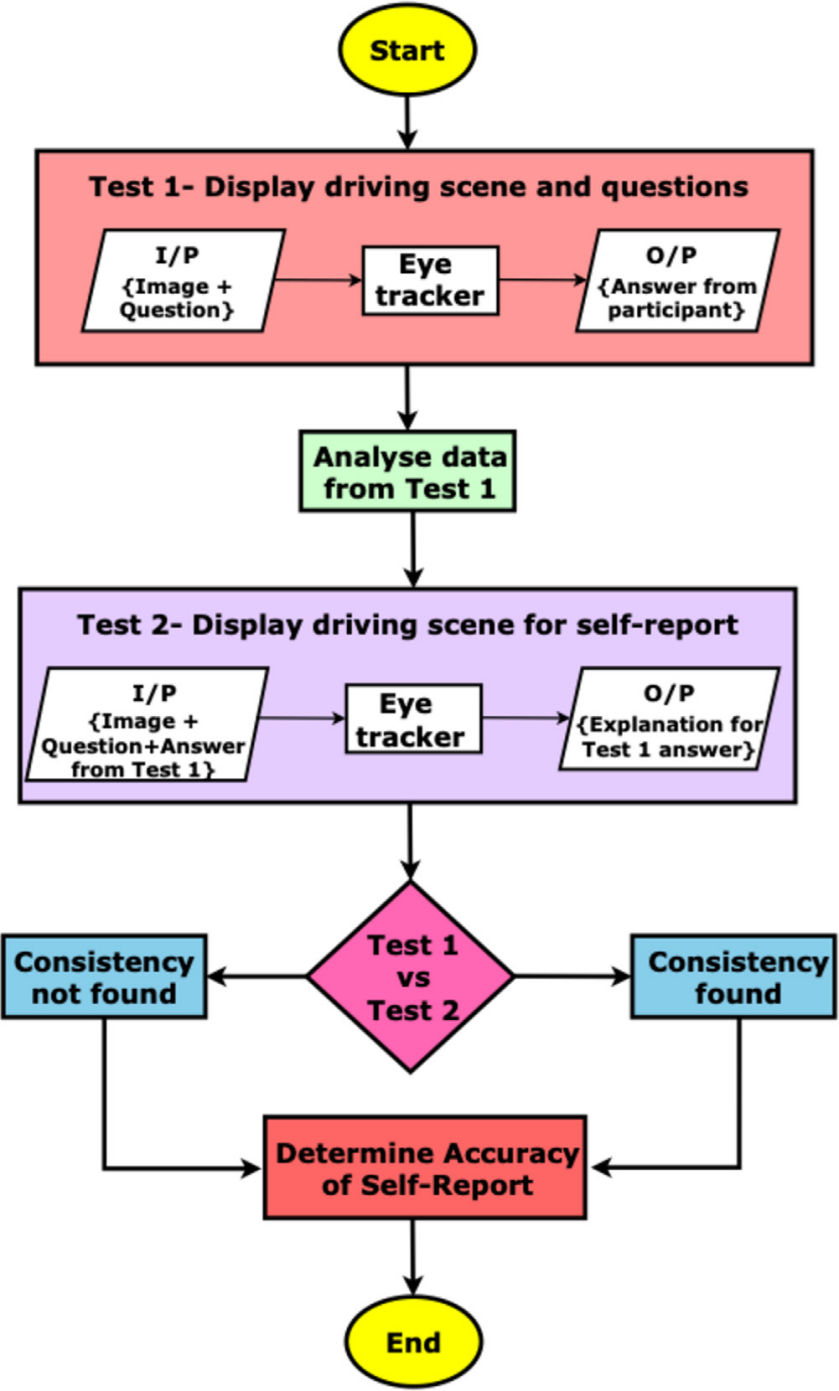
Flowchart illustrating the setup and steps of the eye-tracking experiment for evaluating visual attention in driving scenes.

For the experiment (detailed in Fig. 2), the eye-tracking device to was used to record where a person looks on the screen when a driving scene is presented, and the corresponding question is asked. The experiment began with the presentation of driving scene images, sourced from the NuImages dataset [2], paired with contextual questions (Test 1). Each image was displayed for 5 s, during which an eye-tracking device recorded the participant's gaze data at 5 frames per second (fps) to capture meaningful attention shifts. This data, including gaze plots and heatmaps, was stored for subsequent analysis.

A frame rate of 5 fps was chosen for the eye-tracking setup to balance data quality and practicality. In a driving scenario, key changes in visual attention occur over short but manageable time intervals, and 5 fps is generally sufficient to capture these shifts without overwhelming the system with excessive data. Since human attention movements in driving are typically slower and more deliberate, a higher frame rate would not necessarily provide additional valuable insights. At the same time, it would require more storage and computational resources. Thus, 5 fps allows for efficient data collection and processing while capturing meaningful attention patterns.

In Test 2, participants are shown the same images and asked to recall where they focused their attention during the first test, specifically when answering each corresponding question. This self-reported gaze data is recorded separately. The experiment then progresses to a comparison stage, where Test 1 gaze data is matched against Test 2 self-reports to evaluate consistency.

The decision point in the flowchart (diamond shape) assesses whether the actual gaze data from Test 1 aligns with participants' self-reported focus areas from Test 2. Based on this comparison, participants' responses are classified as “Consistency Found” or “No Consistency” and directed accordingly for further analysis. In both cases, the experiment proceeds to determine the accuracy of participants’ self-reports, which helps to gauge the reliability of subjective responses in relation to objective gaze data. The experiment concludes after this final assessment.

In the dataset provided, there are a total of 24 driving scenarios, each containing five key elements: images of driving situations (24), 48 associated questions (24+24), 1632 participant answers (34×24×2), 1632 gaze plots (816×2), and 1632 heatmaps (816×2). Additionally, there are 48 absolute heatmaps (24×2) and 48 absolute gaze plots (24×2) available for every scenario in both tests. An example of the dataset for a sample question (randomly picked) is shown in Fig. 2.

**Fig. 3:**
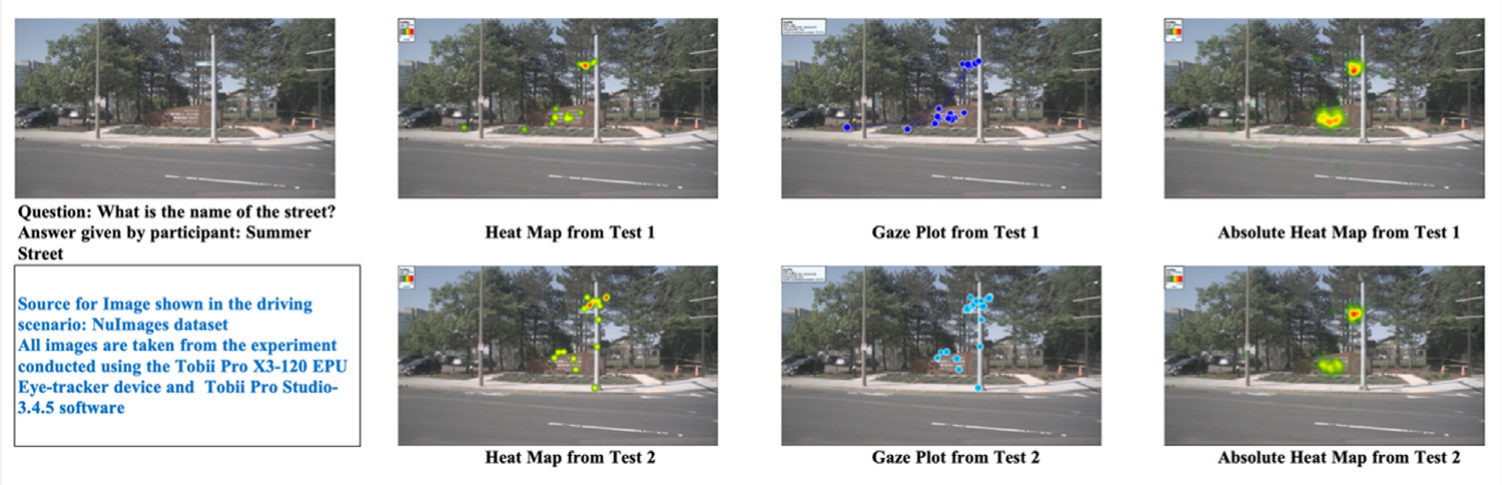
Example of the dataset for a sample question.

All participants in the experiment have a driving experience of a minimum of two (2) years. The participants’ driving experience with their ID has been listed in the document Driving Experience (Fig. 3).

## Limitations

While our sample of 34 participants provides valuable insights, it is acknowledged that it represents a relatively small cohort, which may limit the generalizability of findings to broader populations. To enhance diversity, participants were selected with varying levels of driving experience, as reflected in the driving experience profiles. Additionally, the dataset includes the geographic locations of the participants, providing further context and enhancing the representativeness of our findings.

However, despite these improvements, the sample size remains a limitation. Future studies could benefit from a larger participant pool that encompasses a wider range of age groups, geographic locations, and cultural backgrounds. Expanding the sample size would help verify the consistency of our findings across diverse demographics.

Moreover, while the current dataset offers a meaningful foundation for examining subjective human attention in driving contexts, future research could integrate datasets from similar studies or larger population samples to enhance the robustness and universality of the results. Collaborations with transportation agencies or leveraging data from driving simulators in varied environments could further strengthen the generalizability of these findings.

## Ethics Statement

The relevant informed consent was obtained from the subjects of the data gathering exercise. This research was carried out in accordance with the Declaration of Helsinki and includes the Ethical Committee approval of the University of Limerick (protocol/approval number: 2024_06_06_S&E).

Researchers are mindful of the fact that participants have a right to be protected from public scrutiny of their private lives. To this end, the researcher ensured that all the participants were adequately informed about the objective of this study. In addition, every participant's data stays anonymous and his or her illness states is with the utmost confidentiality.

## CRediT Author Statement

**Kaavya Rekanar:** Conceptualization, Methodology, Software, Data curation, Investigation, Formal Analysis, Visualization, Validation, Writing- Original draft preparation. **John M. Joyce:** Software, Methodology, Resources, Visualization, Formal Analysis, Writing- Reviewing and Editing. **Martin Hayes:** Supervision, Writing- Reviewing and Editing, Project Administration. **Ciarán Eising:** Supervision, Resources, Conceptualization, Writing- Reviewing and Editing, Project Administration, Funding acquisition.

## Data Availability

Mendeley DataDriVQA: A Gaze-Based Dataset for Visual Question Answering in Driving Scenarios (Original data).
